# Performance Enhancement of Polymerized, Functionalized Solution Styrene–Butadiene Rubber Composites Using Oligomeric Resin towards Extremely Safe and Energy-Saving Tires

**DOI:** 10.3390/polym14142928

**Published:** 2022-07-20

**Authors:** Neng Ye, Zhenya Wu, Xiaohui Wu, Yonglai Lu, Liqun Zhang

**Affiliations:** 1 State Key Laboratory of Organic-Inorganic Composites, Beijing 100029, China; ye120neng@163.com (N.Y.); wu_zhen_ya@sina.com (Z.W.); zhanglq@mail.buct.edu.cn (L.Z.); 2 Key Laboratory of Carbon Fiber and Functional Polymers, Ministry of Education, Beijing University of Chemical Technology, Beijing 100029, China; 3 Engineering Research Center of Elastomer Materials Energy Conservation and Resources, Ministry of Education, Beijing University of Chemical Technology, Beijing 100029, China

**Keywords:** tread composite, polymerized functionalized solution styrene–butadiene rubber, oligomeric resin, tire “magic triangle”

## Abstract

Polymerized, functionalized solution styrene–butadiene rubber (F-SSBR) is a new type of polymerized styrene–butadiene rubber solution containing specific terminal groups, which can be used in treads for high performances. However, the wet skid resistance related to safety, the rolling resistance to energy consumption, and the wear resistance to service life are often contradictory and form the performance “magic triangle”. In this work, oligomeric resins, including Coumarone resin, C_9_ resin, C_5_/C_9_ resin and a styrene-α-methyl styrene copolymer (SSC), were used as tire functional additives and selected to replace treated distillate aromatic extract (TDAE) to improve the performances of silica-filled F-SSBR composites. The C_9_ resin, C_5_/C_9_ resin and SSC could enhance the modulus at 300% and tensile strength of the F-SSBR composite. The four resins could improve the wet skid resistance and wear resistance of the composites. However, Coumarone resin caused poor silica dispersion in the F-SSBR matrix and eventually, the lower modulus, higher loss factor at 60 °C and the higher heat buildup in the composite were comparative to the composite with TDAE. Furthermore, the synergistic effect of the C_5_/C_9_ resin and SSC was found to improve the mechanical performance of the composites and it resulted in higher tensile strength and modulus, and a lower heat buildup, compared to the case when only TDAE was used. It is noted that the properties “magic triangle” was broken by the C_5_/C_9_ resin and SSC, and the C_5_/C_9_10T15 increased the wet skid resistance by 21.7%, fuel-saving rate by 2.3%, and wear resistance by 8.3%, while S20T5 increased the wet skid resistance by 30.4%, fuel-saving rate by 7%, and wear resistance by 25% compared with CG.

## 1. Introduction

Polymerized, functionalized solution styrene–butadiene rubber (F-SSBR) is a new type of polymerized solution styrene–butadiene rubber (SSBR) containing specific terminal groups [1,2,3]. These terminal groups can react with the silanol on silica, resulting in better silica dispersion and greater performance of the silica-filled composite [4,5]. Therefore, F-SSBRs have the potential to create high-performance tread [6,7]. Wet skid resistance, rolling resistance and wear resistance are the three key performance areas for tires, but they are often contradictory and form a “magic triangle” of tire performance [8,9,10,11], and F-SSBR also has this problem. Moreover, F-SSBR has poor processing performance and a large amount of treated distillate aromatic extract (TDAE) needs to be compounded, however, adding massive TDAE will deteriorate the performance.

Previously, some studies have demonstrated that a small amount of oligomeric resin used in SSBR is conducive to improving the dynamic mechanical performance of rubber composites, especially the wet skid resistance [12,13,14,15]. This is because the oligomeric resin has a higher glass transition temperature (T_g_) than the rubber matrix [16,17,18]. Moreover, due to its low molecular weight [19,20,21] and low softening point [22,23], the oligomeric resin can improve the processability of composites at a certain processing temperature higher than their softening point. However, F-SSBR was developed in recent years and there is little research on oligomeric resin being used in F-SSBR. The influence of different oligomeric resins on the performances of F-SSBR is still a blank.

In this work, four amorphous resins with a softening point of about 100 °C were selected, including Coumarone resin, C_9_ resin, C_5_/C_9_ resin, and styrene-α-methyl styrene copolymer (SSC). Compared with TDAE, the effects of these resins on silica-filled F-SSBR composites were studied, aiming to fill the research gap in F-SSBR. Moreover, the performances of silica-filled F-SSBR composites containing different amounts of resin (C_5_/C_9_ resin and SSC) were studied and compared to evaluate the potential of the tire functional additives, and thus optimize the “magic triangle” properties of the composites and ensure their good processing properties.

## 2. Experimental Section

### 2.1. Materials

**Figure 1:**
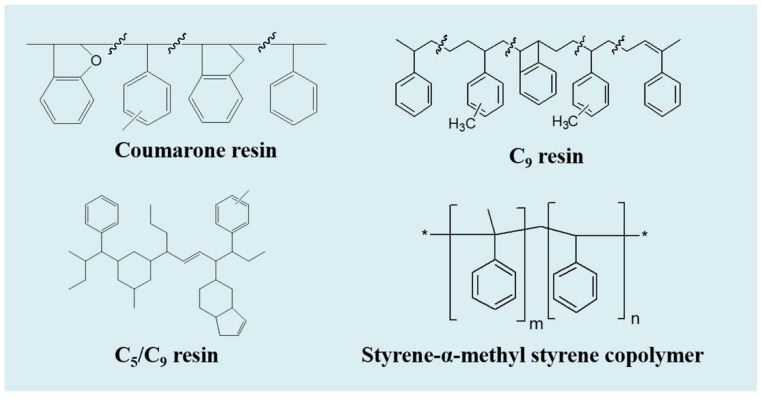
The chemical structures of the four resins.

SSBR SE-0212 was obtained from Sumitomo (Kobe, Japan). Cis-polybutadiene (BR) CB-24 was obtained from Lanxess (Cologne, Germany). Silica VN-3 was supplied by Evonik Degussa (Essen, Germany). TDAE was obtained from TotalEnergies (Paris, France). Coumarone resin, C_9_ resin, C_5_/C_9_ resin and SSC were all obtained from Red Avenue New Materials Group Co., Ltd. (Shanghai, China) and their main performance areas were measured and summarized in Table 1, including number average molecular weight (M_n_), polymer dispersity index (PDI), T_g_, softening point, and density. The chemical structures of the four resins are shown in Figure 1. Bis-(γ-triethoxysilylpropyl)-tetrasulfide, stearic acid, zinc oxide, *N*-tert-butylbenzothiazole-2-sulphenamide, tetramethylthiuram disulfide, *N*-1,3-dimethylbutyl-*N*’-phenyl-*p*-phenylenediamine, wax and sulfur were commercially available.

### 2.2. Preparation of Composites with Resins

In order to study the effect of resin on the silica-filled F-SSBR composite, the samples containing the four different resins were prepared and compared to the control group (CG) that contained the same dosage of TDAE. The formula is displayed in Table 2. To further optimize the properties of the composites, the dosage of C_5_/C_9_ resin and SSC were adjusted, and the weight ratio of resin to TDAE was adjusted to 25:0, 20:5, 10:15, and 5:20. The composites containing C_5_/C_9_ resin or SSC are named C_5_/C_9_xTy and SxTy, respectively, where the x represents the dosage of C_5_/C_9_ resin or SSC resin and y represents the dosage of TDAE. The preparation of composites was carried out in the following steps. Firstly, F-SSBR, BR, TDAE and resin were mixed in internal mixer for 5 min at 110 °C, with a rotor speed of 60 rpm. Then, half of silica and Si69 were added and mixed for 3 min. The other half of silica and Si69 were added and mixed for 3 min. After that, stearic acid, zinc oxide, *N*-1,3-dimethylbutyl-*N*′-phenyl-*p*-phenylenediamine and wax were added and stirred for 3 min. Afterward, the rubber mixture was mixed at 145 °C for 7 min. After the mixer is cooled to room temperature, *N*-tert-butylbenzothiazole-2-sulphenamide, tetramethylthiuram disulfide and sulfur were added and mixed for 5 min. Finally, the compounds were vulcanized at 160 °C to obtain rubber composites. The compounds used for static mechanical properties test were heated at 160 °C for the positive vulcanization time (t_c,90_), and the other test samples were heated for 1.5 times of t_c,90_.

### 2.3. Characterization

The molecular weight and molecular weight distribution of the four resins used in this study were characterized by gel permeation chromatography on a Waters Breeze instrument equipped (Waters Co., Milford, MA, USA) with three water columns (Steerage HT3, HT5, HT6E). The T_g_ of resins was determined by differential scanning calorimetry, using a DSC1 instrument (Mettler Toledo Inc., Zurich, Switzerland). Under a nitrogen environment, the samples were heated from 25 °C to 190 °C at a rate of 10 °C/min and kept for 5 min to eliminate thermal history. Afterward, the temperature was lowered to −50 °C and then increased to 190 °C at the same rate. The softening points of the resins were then tested using a PKA-2 softening point tester (PetroTest Inc., Benson, NC, USA) following the ASTM D36/D36M-09.

Moreover, the vulcanization kinetics of rubber compounds at 160 °C were studied by using an MR-C3 disc rotor vulcanizer (Beijing Ruida Yuchen Instrument Co., Ltd. (Beijing, China) with a rotation frequency of 1.67 Hz and a rotation angle of 0.5°. The viscosities of compounds were measured by using a Mooney Viscometer M3810C (Huanfeng chemical machinery experimental factory, Beijing, China). The rubber compounds with resins were preheated in the viscometer at 100 °C for 1 min and then, the viscosity at 5 min was recorded as ML(1 + 4)100 °C.

A Tecnai G^2^ 20 transmission electron microscope (FEI Co., Hillsboro, OR, USA) was used to observe the microstructures of rubber composites with resins at 200kV. A CMT4104 tensile machine (SANS Test Machine Inc., Shenzhen, China) was used to obtain the engineering stress–strain curves of rubber composites, and the samples were prepared according to ISO 37:2005 specifications. An XSH shore A hardness tester (YingKou material testing machine Co., Ltd., YingKou, Liaoning, China) was used to measure the shore A hardness of the composites. The loss factors (tanδ) of composites, which are defined by the ratio of loss modulus and storage modulus, were characterized by using a VA-300 dynamic mechanical analyzer (01db-Metravib Co., Paris, France) from −40 °C to 100 °C, while the strain was set to 0.1% and the frequency was set to 10 Hz. The heat buildups of the samples were characterized by a YS-Ⅱ Goodrich flexometer (Shanghai ShenRui Testing Machines Inc., Shanghai, China), and the columnar samples were regularly pressed on a load of 25 kg at 55 °C, and the frequency of pressing was 30 Hz. The Akron abrasion was then tested using an MZ-4061 Akron machine (Jiangsu Mingzhu experimental machinery Co., Ltd., Yangzhou, Jiangsu, China) following the BS 903: A9: 1988 (Method B).

## 3. Results and Discussion

### 3.1. Filler Dispersion in the Composites with Different Resins

**Figure 2:**
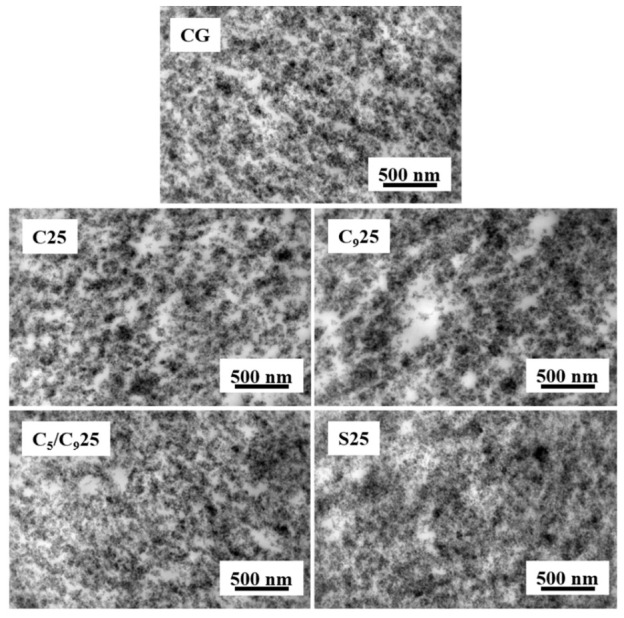
TEM images of composites with different resins.

The mechanical properties of the composites are largely affected by the filler dispersion. The transmission electron microscope (TEM) images of silica-filled F-SSBR composite are shown in Figure 2, in which the gray spot represents the silica in the composite and the dark area represents the silica aggregates. In the TEM image of C_9_25, abundant silica aggregates appeared, which indicates it has the poorest silica dispersion among all composites [24,25,26]. The silica dispersion of CG, C25 and C_5_/C_9_ is similar. Note that more gray areas and only a few dark areas appeared in the TEM image of S25, and its silica dispersion is better than CG. Therefore, it could be seen that the C_9_ resin substituted for TDAE worsens the silica dispersion in F-SSBR, while SSC can promote the silica dispersion in F-SSBR.

### 3.2. Processing Performance and Vulcanization Kinetics of Compounds with Different Resins

**Figure 3:**
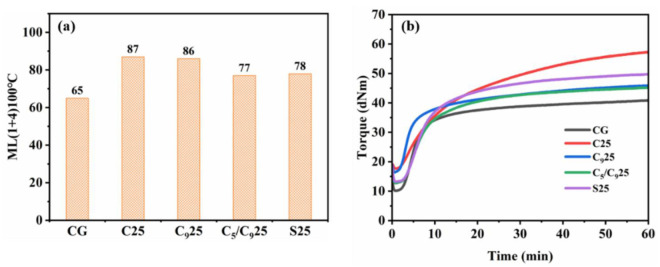
Mooney viscosities (**a**) and vulcanization characteristics curves (**b**) of compounds with different resins.

Mooney viscosity is an index widely used to evaluate the processability of rubber compounds and the lower Mooney viscosity indicates the better fluidity of a rubber compound [27,28,29]. Generally, the better fluidity of rubber compounds means greater plasticity. The Mooney viscosities of compounds with different resins are shown in Figure 3a. When TDAE was replaced with the same dosage of resin, the Mooney viscosities of the compounds increased. The Mooney viscosities of C_5_/C_9_25 and S25 are 77 and 78, respectively, which are the two lowest viscosities, and about 19% higher than that of CG. C25 and C_9_25 have the two highest Mooney viscosities which are about 33% higher than that of CG. Therefore, the plasticizing effect of the C_5_/C_9_ resin and SSC is better than that of the Coumarone resin and C_9_ resin. This is because the C_5_/C_9_ and SSC resin have lower softening points than C9 and Coumarone resin, although SSC has the highest molecular weight among the four resins. In summary, the Mooney viscosity of the compound with the oligomeric resin is affected by both the softening point and the molecular weight of the resins. The lower the softening point and molecular weight of the resin, the better plasticizing effect for the compound, and the C_5_/C_9_ resin has the best plasticizing effect among the four resins.

The vulcanization kinetics of the rubber compounds are studied by using the torque–time curve, as shown in Figure 3b. M_L_, M_H_, M_H_-M_L_, scorching time (t_c,10_), and t_c,90_ are summarized in Table 3, in which the M_L_ of the composites is consistent with the Mooney test results. Generally, the higher M_H_-M_L_ indicates a greater crosslinking network of composites. Note that the M_H_-M_L_ of C_9_25 and S25 is significantly higher than that of CG, indicating that the SSC and Coumarone resin can improve the crosslinking density of the F-SSBR composites. Moreover, the Coumarone resin, C_5_/C_9_ resin and SSC substituted for TDAE prolong the t_c,10_ and t_c,90_ of the compounds, but the t_c,10_ of C_9_25 is shorter than that of CG. Due to the “marching modulus”, C25 has the longest t_c,90_. It is speculated that the Coumarone resin possibly affects the function of the accelerator and activator in the curing system.

### 3.3. Mechanical Performances of the Composites with Different Resins

**Figure 4:**
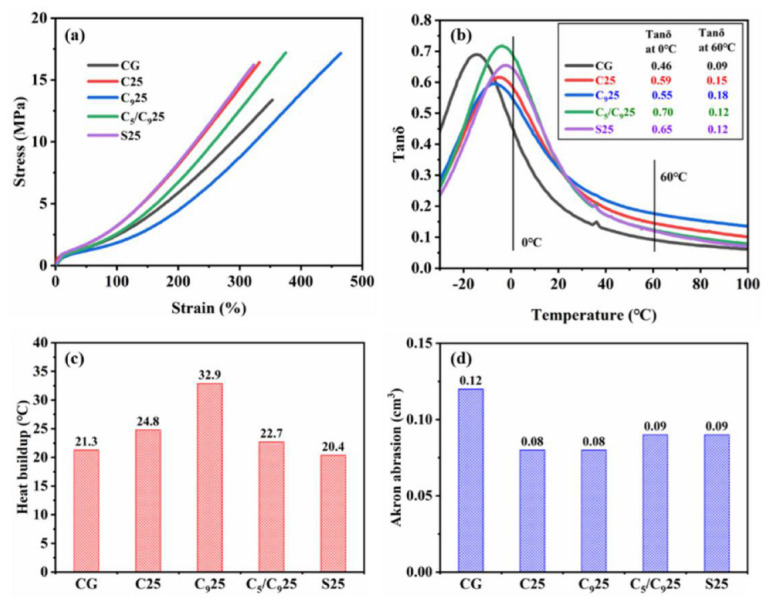
Mechanical performance of the composites with different resins: (**a**) stress–strain curves, (**b**) tanδ–temperature curves, (**c**) heat buildups, and (**d**) Akron abrasions.

Different from TDAE, the T_g_ of the four resins is higher than room temperature (25 °C) and they have higher moduli at room temperature due to their higher softening points, which cause major changes to the static and dynamic mechanical properties of the F-SSBR composites. The static mechanical properties of the composites are characterized by tensile testing instruments and their stress–strain curves are displayed in Figure 4a. The elongation at break, modulus at 100%, modulus at 300%, tensile strength and Shore A hardness of the composites are summarized in Table 4. It could be seen that the composites with the resin have higher tensile strength than CG. It is noted that C25, C_5_/C_9_25 and S25 have higher modulus at 100%, modulus at 300% and Shore A hardness than CG, while C_5_/C_9_25 has the highest tensile strength. However, the C_9_ resin affected the modulus of the composite and the modulus at 300% of C_9_25 is lower than CG. In summary, Coumarone resin, C_5_/C_9_ resin and SSC used as functional additives can effectively improve the modulus at 300% and tensile strength for F-SSBR composites.

The dynamic mechanical performance of the composites was studied, including the wet skid resistance, the rolling resistance, the heat buildup and the wear resistance. The tanδ–temperature curves of composites are shown in Figure 4b, in which the peak of tanδ represents the glass transition of the composite. It is noted that the F-SSBR composites with different resins have only one tanδ peak, which indicates that the four resins have good compatibilities with the F-SSBR matrix. Due to the higher T_g_ of the resins compared with TDAE, the tanδ peaks of the composites containing resins shift to the high-temperature direction. Therefore, C25, C_9_25, C_5_/C_9_25 and S25 have higher tanδ at 0 °C than CG, and this parameter is often positively correlated with the wet skid resistance of tires [30,31,32]. The C_5_/C_9_ resin has the best effect on improving the wet skid resistance of the composites, followed by SSC. Compared to CG, C25, C_9_25, C_5_/C_9_25 and S25 have higher tanδ at 60 °C under the 0.1% strain. The tanδ at 60 °C for C_9_25 is 0.18, which is the highest and indicates that the C_9_ resin has sharply increased the energy loss in the movement of the rubber molecular chain. Among the composites containing resins, the tanδ at 60 °C for C_5_/C_9_25 and S25 is the lowest, and only 0.12. According to Figure 4c, it could be found that the Coumarone resin, C_9_ resin, and C_5_/C_9_ resin have increased the heat buildup of the F-SSBR composites. Due to the large deformation (>5%) of the sample during the heat buildup test, the test can simulate the actual movement of the tire more truly, and further reflect the fuel consumption of the tire. Under the large deformation, the heat buildup of the composites, especially with high filler content, is more contributed to the friction of silica particles. Therefore, the heat buildup of the composite largely depends on the filler dispersion in the sample. The heat buildup in C_9_25 is the largest, which is not at the same level as other samples. This is because the C_9_ resin worsens the silica dispersion in F-SSBRs, resulting in more deformation and friction of the silica aggregates during the repeated compression with the large deformation [33,34]. Note that the heat buildup in S25 is lower than CG because the silica dispersion in S25 is the best. During tire movement, the rubber will cause heat generation and surface damage, and finally abrasion. Akron abrasion is a common method to measure rubber abrasion, which can characterize the wear resistance of rubber composite. The Akron abrasion of the composites, shown in Figure 4d, indicates that the resin can effectively improve the wear resistance of the F-SSBR composite compared to TDAE.

### 3.4. Synergistic Effect of Resin and TDAE for Silica-Filled F-SSBR Composites

According to the above test results, C_5_/C_9_25 and S25 have better static mechanical properties, better wet skid resistance and higher wear resistance than CG, while the heat buildup in S25 is lower than that of CG. However, C_5_/C_9_25 has a higher heat buildup than CG, which means higher energy consumption during tire movement. In order to balance the static and dynamic mechanical properties of silica-filled F-SSBR composites, the composites containing resin (C_5_/C_9_ or SSC) and TDAE were prepared and their dosage ratio was adjusted. Compared to the CG and the composites with only resin, the synergistic effect of resin and TDAE on the processability, vulcanization kinetics and mechanical properties of the F-SSBR composites were studied.

**Figure 5:**
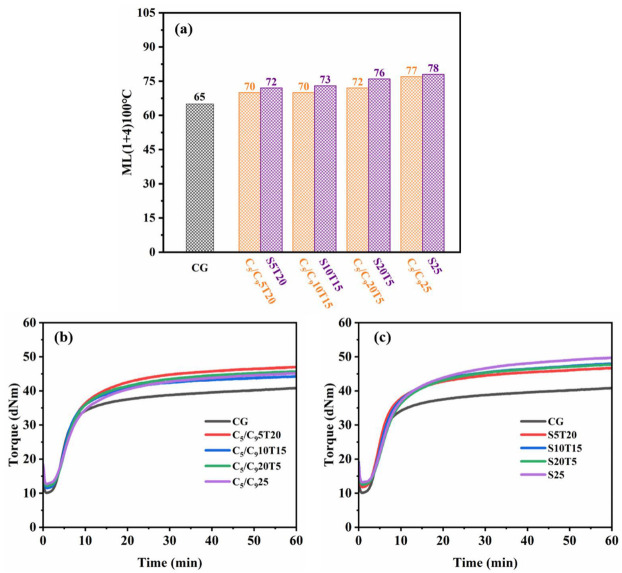
Mooney viscosities of the compounds with resin and TDAE (**a**), and vulcanization characteristic curves of the compounds with C_5_/C_9_ resin and TDAE (**b**) and the compounds with SSC and TDAE (**c**).

As shown in Figure 5a, the Mooney viscosity of the composite increases with the dosage of the C_5_/C_9_ resin or SSC. When the resin is used alone, the Mooney viscosity of the composite is too high, which is not conducive to processing and can be reduced to an appropriate range after compounding TDAE. Figure 5b shows the vulcanization curve of the compound containing resin and TDAE, and summarizes some key vulcanization data in Table 5. The vulcanization kinetics of the composites containing different dosages of the C_5_/C_9_ resin are very similar, with longer t_c,10_ and t_c,90_ compared to CG. The t_c,90_ of the samples with SSC and TDAE is lower than that of S25.

**Figure 6:**
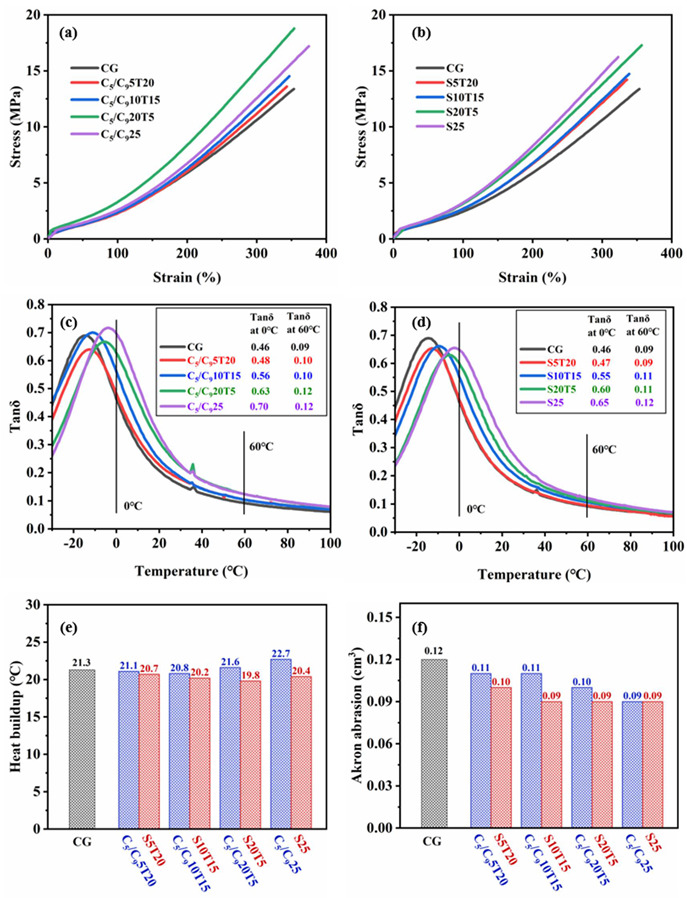
Mechanical performance of the composites with resin and TDAE: (**a**) stress–strain curves of the composites with C_5_/C_9_ resin and TDAE, (**b**) stress–strain curves of the composites with SSC and TDAE, (**c**) tanδ–temperature curves of the composites with C_5_/C_9_ resin and TDAE, (**d**) tanδ–temperature curves of the composites with SSC and TDAE, (**e**) heat buildups, and (**f**) Akron abrasions.

The stress–strain curves of the composites with resin (C_5_/C_9_ or SSC) and TDAE (Figure 6a) indicate the composites with the C_5_/C_9_ resin or SSC have better static mechanical performance than that of CG. Some important mechanical data are summarized in Table 6. It could be seen that the synergistic effect between resin (C_5_/C_9_ or SSC) and TDAE can improve the static mechanical performance of the silica-filled F-SSBR composite. Note that C_5_/C_9_20T5 have the highest modulus at 300% and tensile strength. The modulus at 300% of C_5_/C_9_20T5 is increased by 42% and the tensile strength is increased by 43% compared to those of CG. Moreover, the modulus at 300% of the composites increases with the SSC dosage while S20T5 has the highest tensile strength among the F-SSBR composites containing SSC and TDAE. It is probably because the combination of resin and TDAE not only retains the plasticization of TDAE but also strengthens the functionality of the resin and has a stronger reinforcing effect.

The tanδ–temperature curves (Figure 6b) indicated that the composite with more C_5_/C_9_ resin or SSC has a higher tanδ at 0 °C and 60 °C. The heat buildups are displayed in Figure 6c. S20T5 has the lowest heat buildup, which indicates the greatest fuel saving. Among the composites with the C_5_/C_9_ resin and TDAE, the heat buildups of C_5_/C_9_5T20 and C_5_/C_9_10T15 are lower than that of CG, and C_5_/C_9_10T15 has the lowest heat buildup. It proves that the synergistic effect between resin (C_5_/C_9_ or SSC) and TDAE can reduce the heat buildup of the silica-filled F-SSBR composite. The Akron abrasions of the composites are shown in Figure 6d and it could be found that composites containing more C_5_/C_9_ resin or SSC have higher wear resistance.

### 3.5. Balance of Dynamic Performance for F-SSBR Composites with Resin

**Figure 7:**
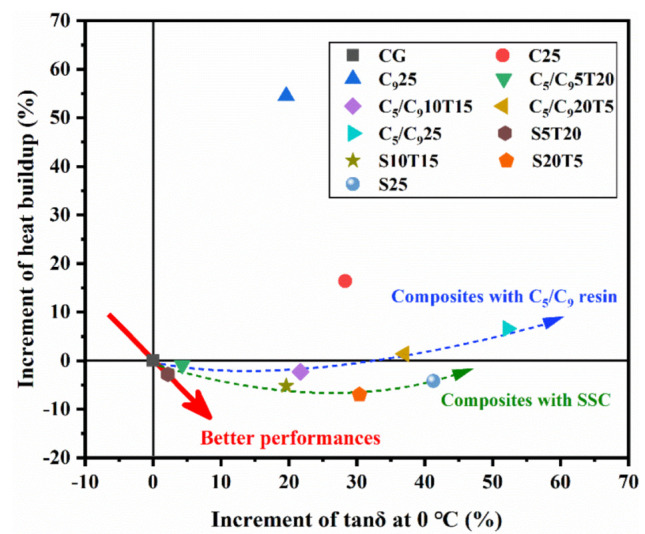
Balance between heat buildup and tanδ at 0 °C for F-SSBR composites. The increment is the proportion of difference between samples and CG in CG.

Generally, rolling resistance and wet skid resistance are two antagonistic dynamic performances of a tire. In order to study the effect of resin type and its amount on the two dynamic performances of silica-filled F−SSBR composites, a scatter diagram according to the increment of the heat buildup and the tanδ at 0 °C of each sample is shown in Figure 7, while the dynamic performance of CG was used as a reference. The increment is calculated according to the following two formula:

Increment of heat buildup = [Heat buildup (CG) − Heat buildup (Sample)]/Heat buildup (CG) × 100%

Increment of tanδ = [tanδ (Sample) − tanδ (CG)]/tanδ (CG) × 100%

The composites located in the fourth quadrant have better properties, including C_5_/C_9_5T20, C_5_/C_9_10T15, S5T20, S10T15, S20T5 and S25, which represent a simultaneous enhancement of the fuel-saving and the wet skid resistance of the F-SSBR composites. Compared to CG, the heat buildup of C25 and C_9_25 increased significantly although their tanδ at 0 °C was improved. The increments of tanδ at 0 °C for C_5_/C_9_25 and S25 are higher than that of C25 or C_9_25, while their heat buildups are not significantly increased and with S25 it is even reduced. Note that the properties of the F-SSBR composites are becoming better with increases in the C_5_/C_9_ resin or SSC and then becoming worse, which indicates that there is an optimal dosage.

**Figure 8:**
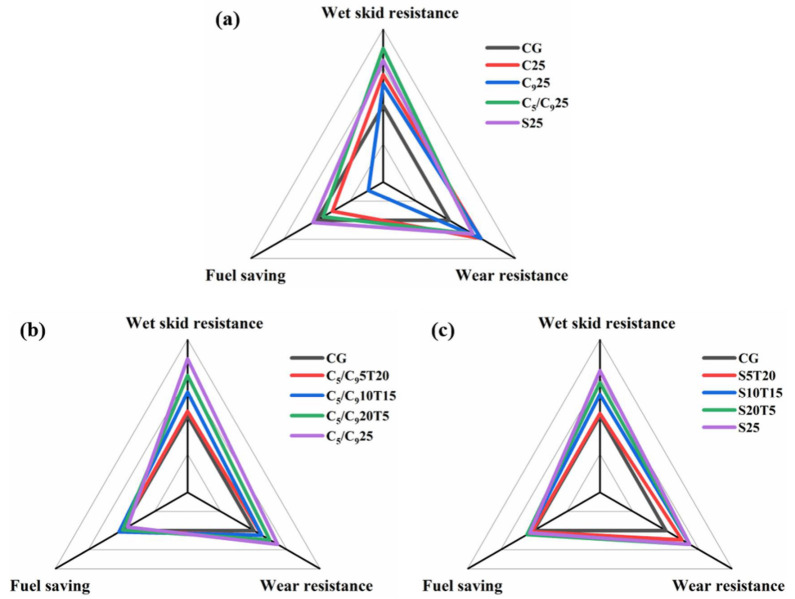
“Magic triangle” of F-SSBR composites with four different resins (**a**), with C5/C9 resin and TDAE (**b**), and with SSC and TDAE (**c**). Each scale increment is 35%, representing the performance enhancement.

In summary, the C_5_/C_9_ resin and SSC could greatly improve the static mechanical properties, wet skid resistance and wear resistance of F-SSBR composites. It means that the resins in the F-SSBR composites affect the performance “magic triangle”, as shown in Figure 8. Therefore, the dosage of resins in the F-SSBR composites needs to be adjusted in order to balance the “magic triangle” performance of the tread. Note that the C_5_/C_9_ resin and SSC could break the properties “magic triangle”, and the silica-filled F-SSBR composites with resin (C_5_/C_9_ or SSC) and TDAE could simultaneously achieve a better wet skid resistance, fuel-saving, and wear resistance, which proves their potential for tire functional additives. Considering the balance of the “magic triangle” properties, C_5_/C_9_10T15 and S20T5 were better choices. Compared with CG, C_5_/C_9_10T15 increased the wet skid resistance by 21.7%, fuel-saving rate by 2.3%, and wear resistance by 8.3%, while S20T5 increased the wet skid resistance by 30.4%, fuel-saving rate by 7%, and wear resistance by 25%.

## 4. Conclusions

Coumarone resin, C_9_ resin, C_5_/C_9_ resin and SSC with a lower softening point and higher T_g_ were selected to replace TDAE to prepare silica-filled F-SSBR composites. For the static mechanical performances, the C_9_ resin, C_5_/C_9_ resin, and SSC could enhance the modulus at 300% and the tensile strength of the composite. Moreover, the four resins improved the wet skid resistance and wear resistance of the composites. S25 and C_5_/C_9_25 have better dynamic and static properties than the composites with the other two resins, but their processing properties were reduced by 19% compared to CG. Furthermore, the synergistic effect of resin (C_5_/C_9_ or SSC) and TDAE was found to improve the mechanical performance of the composites and it resulted in higher tensile strength and modulus, and a lower heat buildup, compared to the case when only TDAE or resin was used. According to the increment on tanδ at 0 °C and heat buildup, it could be seen that the C_5_/C_9_ resin and SSC were the effective functional additives for a high-performing F-SSBR tread. Note that the C_5_/C_9_ resin and SSC could break the properties “magic triangle”, and C_5_/C_9_10T15 increased the wet skid resistance by 21.7%, fuel-saving rate by 2.3%, and wear resistance by 8.3%, while S20T5 increased the wet skid resistance by 30.4%, fuel-saving rate by 7%, and wear resistance by 25% compared with CG.

## Figures and Tables

**Table 1:** Main performance areas of resins.

	M_n_	PDI	T_g_	Softening Point	Density
	(g/mol)		(°C)	(°C)	(g/cm^3^)
Coumarone resin	1800	1.6	42.0	100	0.91
C_9_ resin	1236	1.2	50.9	117	0.92
C_5_/C_9_ resin	1479	1.3	40.5	90	1.08
Styrene-α-methyl styrene copolymer	2098	1.8	37.1	85	0.95

**Table 2:** Formula of the compounds with different resins ^a^.

Ingredient	Control Group (CG)	C25	C_9_25	C_5_/C_9_25	S25	C_5_/C_9_xTy	SxTy
SSBR SE-0212	70						
BR CB-24	30						
Silica	70						
TDAE	25	0	0	0	0	20/15/5	20/15/5
Coumarone resin	0	25	0	0	0	0	0
C_9_ resin	0	0	25	0	0	0	0
C_5_/C_9_ resin	0	0	0	25	0	5/10/20	0
Styrene-α-methyl styrene copolymer	0	0	0	0	25	0	5/10/20
Other additives ^b^	-						

^a^ Unit is in part per hundred part of rubber (phr); ^b^ Other additives contain 5.6 phr bis-(γ-triethoxysilylpropyl)-tetrasulfide (Si69), 3 phr stearic acid, 2 phr zinc oxide, 2 phr *N*-1,3-dimethylbutyl-*N*’-phenyl-*p*-phenylenediamine, 1.5 phr wax, 1.8 phr *N*-tert-butylbenzothiazole-2-sulphenamide, 0.3 phr tetramethylthiuram disulfide and 2.3 phr sulfur.

**Table 3:** Vulcanization data of compounds with different resins.

	The Lowest Torque (M_L_)	The Highest Torque (M_H_)	M_H_-M_L_	t_c,10_	t_c,90_
	(dNm)	(dNm)	(dNm)	(min:s)	(min:s)
CG	10	41	31	3:04	21:39
C25	18	57	39	3:23	40:29
C_9_25	16	46	30	2:19	30:49
C_5_/C_9_25	13	45	32	3:31	24:28
S25	13	50	37	3:48	27:22

**Table 4:** Static mechanical performance of composites with different resins.

	Elongation at Break	Modulus at 100%	Modulus at 300%	Tensile Strength	Shore A Hardness
	(%)	(MPa)	(MPa)	(MPa)	
CG	353 ± 29	2.4 ± 0.1	10.6 ± 1.0	13.4 ± 2.1	63 ± 1
C25	332 ± 27	3.2 ± 0.2	14.3 ± 0.6	16.4 ± 2.3	67 ± 1
C_9_25	465 ± 43	1.8 ± 0.2	8.8 ± 0.7	17.2 ± 1.2	67 ± 3
C_5_/C_9_25	375 ± 37	2.6 ± 0.3	12.5 ± 1.1	17.2 ± 1.4	64 ± 3
S25	323 ± 31	3.2 ± 0.1	14.7 ± 0.2	16.2 ± 2.1	66 ± 2

**Table 5:** Vulcanization data of compounds with resin (C_5_/C_9_ or SSC) and TDAE.

	The Lowest Torque (M_L_)	The Highest Torque (M_H_)	M_H_-M_L_	t_c,10_	t_c,90_
	(dNm)	(dNm)	(dNm)	(min:s)	(min:s)
CG	10	41	31	3:04	21:39
C_5_/C_9_5T20	12	47	35	3:27	23:21
C_5_/C_9_10T15	11	44	33	3:19	21:45
C_5_/C_9_20T5	12	46	34	3:34	23:47
C_5_/C_9_25	13	45	32	3:31	24:28
S5T20	12	47	35	3:12	21:50
S10T15	12	48	36	3:31	24:07
S20T5	13	48	35	3:42	23:52
S25	13	50	37	3:48	27:22

**Table 6:** Static mechanical performance of composites with resin (C_5_/C_9_ or SSC) and TDAE.

	Elongation at Break	Modulus at 100%	Modulus at 300%	Tensile Strength	Shore A Hardness
	(%)	(MPa)	(MPa)	(MPa)	
CG	353 ± 29	2.4 ± 0.1	10.6 ± 1.0	13.4 ± 2.1	63 ± 1
C_5_/C_9_5T20	343 ± 20	2.3 ± 0.1	11.2 ± 2.3	13.6 ± 2.8	65 ± 3
C_5_/C_9_10T15	347 ± 22	2.4 ± 0.1	11.8 ± 0.2	14.5 ± 1.4	64 ± 2
C_5_/C_9_20T5	354 ± 42	3.3 ± 0.3	15.1 ± 2.0	18.8 ± 0.2	64 ± 1
C5/C925	375 ± 37	3.2 ± 0.3	12.5 ± 1.1	17.2 ± 1.4	64 ± 3
S5T20	336 ± 21	2.7 ± 0.1	12.1 ± 0.3	14.2 ± 2.2	64 ± 3
S10T15	339 ± 43	2.7 ± 0.1	12.4 ± 0.5	14.7 ± 1.7	67 ± 1
S20T5	357 ± 30	3.1 ± 0.4	13.8 ± 1.7	17.3 ± 2.1	67 ± 2
S25	323 ± 31	3.2 ± 0.1	14.7 ± 0.2	16.2 ± 2.1	66 ± 2

## Data Availability

Not applicable.
